# Long term measures of vestibulo-ocular reflex function in high level male gymnasts and its possible role during context specific rotational tasks

**DOI:** 10.1371/journal.pone.0243752

**Published:** 2020-12-14

**Authors:** Christoph von Laßberg, Jennifer L. Campos, Karl A. Beykirch

**Affiliations:** 1 Department Sports Medicine, Medical Clinic, University of Tübingen, Tübingen, Germany; 2 Toronto Rehabilitation Institute–University Health Network, Toronto, Canada; 3 Department of Psychology, University of Toronto, Toronto, Canada; 4 Max Planck Institute for Biological Cybernetics, Tübingen, Germany; University of Alberta, CANADA

## Abstract

In a prior publication, we described a previously unknown eye movement phenomenon during the execution of actively performed multiaxial rotations in high level gymnasts. This phenomenon was consistently observed during the phase of fast free flight rotations and was marked by a prolonged and complete suppression of nystagmus and gaze stabilizing “environment referenced eye movements” (EREM; such as the vestibulo-ocular reflex, optokinetic reflex, smooth pursuit and others). Instead, these eye movements were coupled with intersegmental body movements. We have therefore called it “spinal motor-coupled eye movements” (SCEM) and have interpreted the phenomenon to likely be caused by anti-compensatory functions of more proprioceptive mediated reflexes and perhaps other mechanisms (e.g., top-down regulation as part of a motor plan) to effectively cope with a *new-orientation* in space, undisturbed by EREM functions. In the phase before landing, the phenomenon was replaced again by the known gaze-stabilizing EREM functions. The present study specifically evaluated long-term measures of vestibulo-ocular reflex functions (VOR) in high level gymnasts and controls during both passively driven monoaxial rotations and context-specific multiaxial somersault simulations in a vestibular lab. This approach provided further insights into the possible roles of adaptive or mental influences concerning the VOR function and how they are associated with the described phenomenon of SCEM. Results showed high inter-individual variability of VOR function in both gymnasts and controls, but no systematic adaptation of the VOR in gymnasts, neither compared to controls nor over a period of three years. This might generally support the hypothesis that the phenomenon of SCEM might indeed be driven more by proprioceptively mediated and situationally dominant eye movement functions than by adaptative processes of the VOR.

## Introduction

The population of *high level gymnasts* is unique with respect to the exceptional precision of orientation and motor control they require during fast multiaxial rotations around different axes. These abilities have been systematically acquired by intense daily training over years. Thus, investigating eye movements in this specific population may give further insight into specific adaptive or functional mechanisms of the visual-vestibular system that enable gymnasts to develop their exceptional spatial orientation abilities.

**Fig 1:**
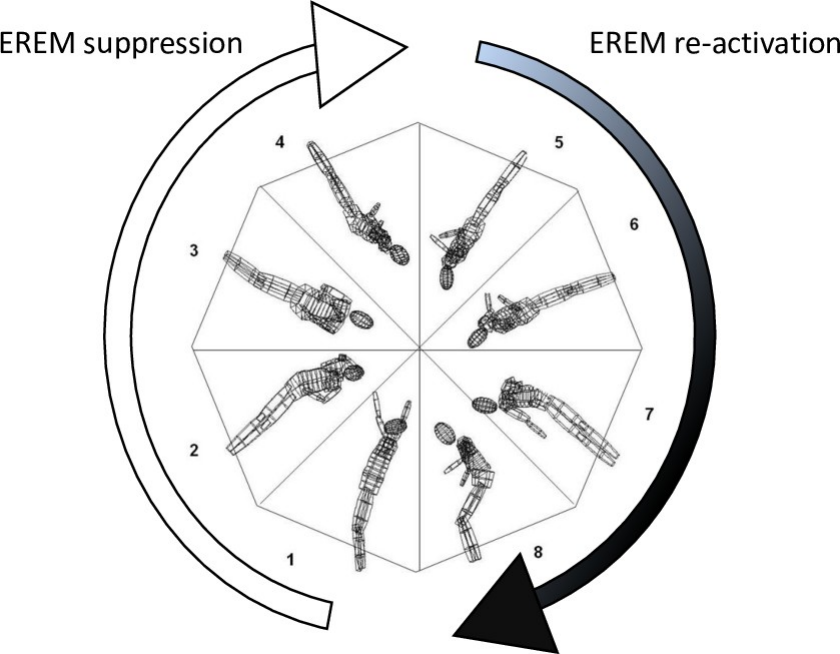
The phenomenon of SCEM. During complete suppression of EREM functions (sectors 1–4) eye movements have been found to be co-directed with head and body movement (SCEM). During the beginning of the preparation for the landing phase (mostly beginning in sectors 5 or 6), the usual compensatory EREM functions are re-activated.

The measurement of vestibularly-mediated eye movement characteristics is typically done with standardized tests during *passive* rotations of the head or whole body. Even though this approach has the essential advantage that specific eye movement functions (for example the VOR) can be *separately* measured under controlled conditions, this also reveals the main disadvantage of passive rotational tests, i.e., they do not take into account additional influences that might be essential during *actively* performed movements. Therefore, in a prior study we investigated for the first time the way oculomotor functions work together with spinal motor and kinematic information during *actively* performed multiaxial free-flight rotations (multiple twisting somersaults) in high level gymnasts [1]. During these measurements in all the gymnasts, a phenomenon was observed that had not been described before. During the phase of fast body rotations, all “environment referenced eye movement functions” (EREM functions) that serve compensatory gaze stabilization of environmental objects during movements (vestibulo-ocular reflex [VOR], optokinetic reflex [OKR], Smooth pursuit [SP] etc.) were *completely suppressed*, and instead, the eye movements were *co-directed* with the body movements during this phase. We therefore called this phenomenon “spinal motor-coupled eye movements” (SCEM) and interpreted this phenomenon to most likely be caused by *anti-compensatory* functions of the more *proprioceptive* mediated reflexes [1] and perhaps top-down control and suppression to most effectively support a *new-orientation* in space, undisturbed by any EREM functions. However, this phenomenon of co-directed (anti-compensatory) SCEM was subsequently replaced by the *compensatory* EREM functions to support the necessary visual control for a safe landing (compare [1]: Videos 1 and 3). We therefore hypothesized that to effectively cope with *actively performed head and body rotations*, eye movements are marked by two counteracting phases with different requirements (Fig 1): a phase that serves to support an effective *new-orientation* of the body in space (by the *anti-compensatory* functions of the SCEMs), and a phase that serves to support oculomotor *re-stabilization* to environmental objects (by the *compensatory* functions of EREMs).

Presumably, the latter phase (supported by EREMs) requires a high level of precision to sufficiently perceive the environmental objects, particularly after very fast and complex rotational movements. This may be consistent with other studies that postulate that the smooth pursuit and optokinetic reflexes during somersault movements are most relevant in providing sufficient visual control to ensure safe landings [e.g., 2–8]. It might therefore be assumed that the EREMs in gymnasts need to be *more accurate* compared with persons who are not required to perform such complex multiaxial maneuvers. In our own studies [9], we found support for this assumption by showing significantly higher performance of the *smooth pursuit function* in gymnasts compared to controls, which was also related to their training schedule. Specifically, after a 3-week training break, the smooth pursuit gain had significantly decreased within the athlete group.

Based on these results and considerations, the question remains which specific role the *VOR* plays during such complex rotational tasks and whether any kind of VOR adaptation might be found in high level gymnasts to help support their exceptional spatial orientation abilities. Even though a complete *suppression* of nystagmus activity was found during the SCEM phases (this necessarily implicates a complete suppression of the VOR), a context-specific *enhancement* of EREM functions (including the VOR) might ensure necessary gaze stabilization during EREM-phases (i.e., before landing).

### VOR characteristics in healthy persons and expert populations

The parameters typically used to describe the dynamic characteristics and precision of the rotational VOR are the values of *VOR-gain* and *VOR-phase* during standardized procedures (e.g., sinusoidal, impulse, and constant velocity tests). The rotational *VOR-gain* is defined as the quotient of eye-velocity and head-velocity during rotational motion in the dark. The VOR-gain is normally measured during passively driven head or whole body movements. The maximum healthy gain value is 1, in which case there is complete compensation of rotational head movements by eye movements. The *VOR-phase* is a measure of the phase shift of the compensatory eye movements compared to a sinusoidal stimulus.

Whereas the phenomenon of per- and postrotatory VOR *habituation* in subjects accustomed to strong rotations has been well documented [e.g., 10–15], the relationships concerning more *context-specific* VOR characteristics seem to be inconsistent and not entirely clear. For instance, Lee et al. [16] reported significantly *higher* VOR gains in a group of fighter pilots than in a group of non-pilots in sinusoidal testing after both groups had the same flight training. Similar findings with significant *increases* of VOR-gains in student pilots vs. non-pilots were reported by Schwarz and Henn [13]. However, *no* gain enhancements of pilots vs. non-pilots were found by Ahn [17]. Tanguy et al. [18] report significantly *lower* gain values among *figure skaters* compared to controls. Clement et al. [15] found *no changes* in VOR-gains after repeated vestibular training (velocity steps) of *normal healthy persons*. Therefore, it has been suggested that VOR adaptations may be caused by highly *context specific cues*, however, the detailed mechanisms responsible for such contrary findings have not yet been completely identified [e.g., 18, 19].

Very few studies have described standardized VOR characteristics *in gymnasts*. Quarck & Denise [20] describe VOR *habituation* in gymnasts, measured during horizontal velocity step tests. Stangl et al. [21] compared horizontal, vertical and torsional VOR gains of male gymnasts (n = 7) to athletes of other sports (ballet dancers and volleyball players) and controls, measured during earth vertical sinusoidal tests. Results were inconsistent concerning the different rotational planes within and between the groups and therefore hard to interpret.

### Aim of the current study

Motivated by the aforementioned paucity of research on VOR characteristics in gymnasts, the current study addressed these gaps by long-term measures in a multiaxial vestibular research stimulator, measuring 1) *standardized* sinusoidal tests and 2) *more context-specific*, *multiaxial* sinusoidal tests (similar to twisting somersaults) under different mental conditions.

The measurements might be able to more clearly evaluate a) whether or not differences between high level gymnasts and controls are observed, b) to what extend mental factors, associated with context-specific loads, influence VOR responses and c) how far VOR characteristics change over a long-time period of three years in gymnasts vs. controls, to more precisely evaluate long-term adaptations in the VOR development during a gymnastics career. This approach addresses open questions concerning specific kinds of training-dependent VOR adaptation and, in particular, the functional role of VOR characteristics with regard to the phenomenon of SCEM.

## Methods

### Ethics statement

All measurements were conducted according to the principles expressed in the Declaration of Helsinki and were undertaken with the understanding and written informed consent of each subject or their parents, in case of juvenile participants. This includes consent for publication of their photograph, as outlined in the PLOS consent form for publication in a PLOS journal. The study was supported by the German Institute for Sport Science and was approved by a decision of the German Parliament. It was not required to obtain approval by an additional institutional review board for this study. This was confirmed by a written waiver of the German Institute for Sports Science. No research was conducted outside of our country of residence.

### General approach and study design

The study consisted of two main parts (see Table 1):

1. A combined *cross-sectional and longitudinal design* included a group of high-level gymnasts (Gym) compared to an age-matched control group of non-athletes (NA) who were tested twice over a period of three years at measurement times T1 and T2. This part of the study consisted of passive sinusoidal whole body rotations in the earth horizontal plane and earth vertical plane (at T1 and T2) and also included combined superimposed multiaxial sinusoidal movements (twisting somersault simulation) at T1 (see section 2.4 for details). Technical problems prevented repeating the multiaxial movements at T2. Therefore, only the monoaxial tests were repeated after three years. Both groups (Gym and NA) were reduced by one participant each at T2. The remaining group of gymnasts continued with their training and even increased their training schedules; the remaining group of non-athletes had not started any intense training in sports during this period.
2. A comparison of the data from the cross-sectional and longitudinal design (Gym and NA) with data from top-level gymnasts (TopGym) to give further insight for tendencies of additional relationships between the group´s level of performance and individual VOR characteristics. The TopGym group, however, was not incorporated in the cross sectional and longitudinal design described above because of age differences with the Gym and NA groups, and an inability to plan participation in such a design due to scheduling difficulties.

**Table 1:** Synopsis of the general design.

	1. Cross-sectional and longitudinal design of high-level gymnasts (Gym) vs. non-athletes (NA)		2. Comparison with top-level gymnasts (TopGym)
Measuring times	T1	T2 (3 years after T1)	(T2)
**Groups**	**Gym; NA**	**Gym; NA**	**TopGym (compared to Gym and NA)**
**Monoaxial sinusoidal tests**	• **Horizontal sinusoidal tests**	• **Horizontal sinusoidal tests**	• **Horizontal sinusoidal tests**
	(Test 1–3 horizontal)	(Test 1–3 horizontal)	(Test 1–3 horizontal)
	• **Vertical sinusoidal tests**	• **Vertical sinusoidal tests**	• **Vertical sinusoidal tests**
	(Test 1–3 vertical)	(Test 1–3 vertical)	(Test 1–3 vertical)
**Multiaxial sinusoidal tests**	• **Twisting somersault tests**	--	--
	(Test 4–5)		

### Participants

The participants of the cross-sectional and longitudinal study were composed of a group of male D-level gymnasts (Gym: N = 9; ages 10–13 years, with a training schedule (TS) of 20 hrs/week at T1. At T2 the group was reduced to N = 8; subjects aged 13–16 years at T2 and the TS increased to 25 hrs/week. An age-matched control group consistent of male non-athletes who did not practice any sports regularly (NA; N = 10, ages: 11–13 years at T1; at T2: N = 9; ages: 14–16 years). The group of top-level gymnasts consisted of male A and B-level gymnasts (TopGym: N = 9; ages: 17–25 years; TS: 25–32 hrs/week).

D-Level gymnasts are defined as a selection of the best regional or state junior gymnasts. A-Level gymnasts are official members of the German National Team (two were ranked within the Top 10 in World Cup competitions). B-Level gymnasts represent those who are at the entry-level of the National Team. All gymnasts were recruited from the National Training Centre for Artistic Gymnastics in Stuttgart, Germany, and competed in all of the six male Olympic gymnastics apparatuses (floor exercise, high bar, parallel bars, vault, pommel horse and rings).

### Apparatus

**Fig 2:**
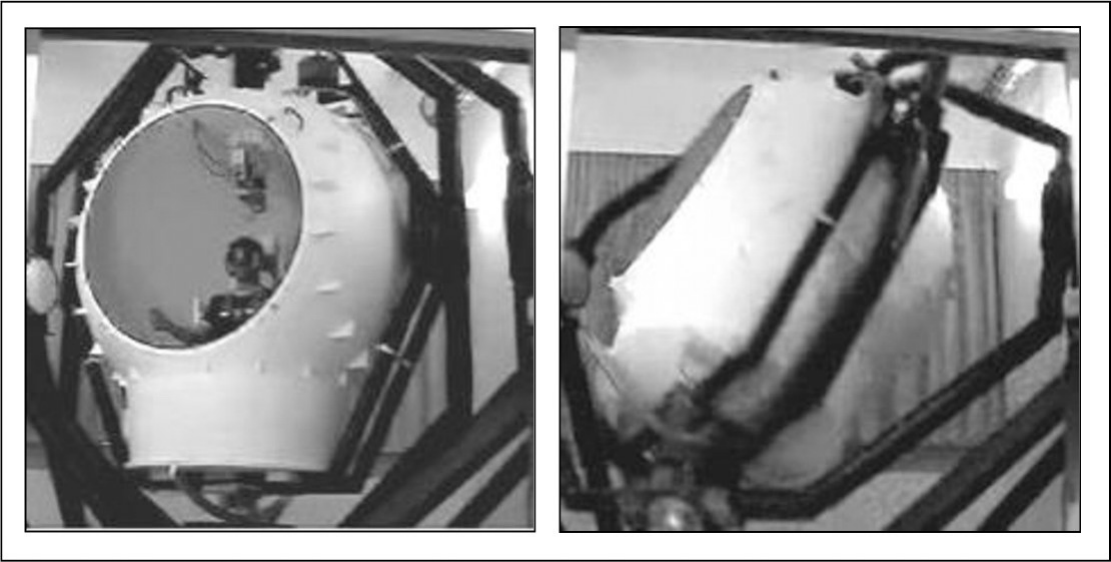
The Tübingen Vestibular Research Stimulator.

All tests were conducted in the Tübingen Vestibular Research Stimulator (TVRS), a multi-axial vestibular stimulation device at the University of Tübingen, Germany (Fig 2). This device was developed as a specific prototype for neurophysiological research [22, 23].

This spherical chamber has a diameter of about 3m and can be moved separately about all rotational axes and can also produce combined movements about two pre-selected axes without an angular limit. It is driven by two computer controlled electric motors. A seat with a five-point harness is mounted in the center of the chamber. The participant’s head is fixed by a head rest and the rotation axis was adjusted exactly to the middle of the head. Oculomotor data was collected and processed via a customized version of a videonystagmography system with wireless data transfer (see later in the text).

### Test protocol and procedure of the measurements

For all the tests, participants were secured in the chair of the TVRS with the five-point harness and their heads were fixed in the headrest to prevent it from moving. The participants wore the videonystagmography mask and were instructed to keep their eyes open and to look “straight forward into the dark” during the tests.

The *sinusoidal tests* included sets of 3 *monoaxial tests* in both the horizontal (Test 1–3 hor) and vertical plane (Test 1–3 vert), always at 0.1 Hz: Test 1 with maximum velocity (Vmax) of 25°/s (39.8°, 15.7°/s^2^); Test 2 with Vmax of 50°/s (79.6°, 31.4°/s^2^); Test 3 with Vmax: 100°/s (159.2°, 62.8°/s^2^). A 30 second pause was introduced between all the tests. At T1, two additional *multiaxial* sinusoidal tests (Test 4–5) were carried out, that consisted of *superposed transversal/longitudinal body axis rotations* (twisting somersaults) (0.1 Hz; 180°; 113.1°/s; 71.1°/s^2^). These tests simulated single twisting somersault movements (one transversal axis rotation [TAR] superposed by one longitudinal axis rotation [LAR]) and were selected to represent maneuvers that are commonly experienced by gymnasts in order to evaluate if there is any context-specific adaptation in the expert group, compared to the controls. Both the TAR and LAR components were programmed as a single sinusoidal oscillation, each beginning turning backward and to the right (first half of the oscillation), followed by turning forward and to the left (second half of the oscillation). These multiaxial sinusoidal tests were conducted twice in an identical manner (with 30 seconds pause between the tests). However, during the first trial (Test 4), the specific kind of the movement was *not described* to the subjects beforehand; during the second trial (Test 5), subjects were instructed that the identical movement would follow again (thus, in contrast to Test 4, participants could anticipate the movement of Test 5).

### Capturing and processing of oculomotor data

The analysis of oculomotor data was based on an infrared camera which captured the left eye movements of the test participants at a frequency of 50 Hz in darkness. In addition to the internal system software (2D VOG, version 3.2, created by SensoMotoric Instruments [SMI]), custom software written using the LabView development environment (National Instruments) was used to calculate the oculomotor parameters. All signals were recorded at 50Hz and sample-rate converted to create an oversampled version that was filtered with a 21-tap-finite-impulse response digital low-pass filter (42Hz cutoff) that was optimized for noise reduction and preservation of critical fast components dynamics [24]. Fast nystagmus components were removed and replaced with interpolated values. The fast component removal software used a phase space plot of response versus stimulus to remove phase and interactively put boundaries around the slow component plot. Values outside the boundaries were removed. Statistical analyses of the noise of the residuals from the best-fit of the slow component response to the stimulus were used to allow for an objective evaluation of the interactive choices. The gain values were obtained from a cross-spectral density of slow component responses to the stimulus at the frequency of the stimulus. Using the analysis software, it was possible to calculate the average VOR gain and use this for further statistical analyses. The evaluation procedure described is one of the most popular and well-established methods in the field of oculomotor research [25, 26]. Although all amplitude values were carefully calibrated, variability in the relative acquisition timing of the stimulus and response caused variability in phase measures. Since the goals of this work were not dependent on phase comparison, phase is not reported.

### Statistical analysis

VOR gains were analyzed by calculating mean values (MV), standard deviations (SD) and coefficients of variance (CoV). Effect sizes were calculated using Cohen’s d [d = (mean x–mean y) / mean SD]. Values of 0.2 < d < 0.5 represent small effects, 0.5 < d < 0.8 represent moderate effects and values > 0.8 represent large effects [27]. To test the stability of values over the different testing conditions and testing times within the same groups, Pearson correlation coefficients were calculated. Fisher's z-transformation procedure (and re-transformation) was used for calculating averaged values.

For inferential analyses, double-sided student t-tests were conducted for both the cross-sectional (independent samples) and the longitudinal (paired samples) data sets. We further applied a linear mixed model for overall mean estimation to obtain an unbiased test statistic. This was based on the fact that the design includes multiple measurements for each test person and the different test conditions. Hence, we do not have independent observations, and standard inferential tests may lead to biased test statistics. Finally, a post-hoc Bonferroni correction was applied. Residual analysis was performed to test the normality assumption of the linear mixed models. The alpha level was set at p = 0.05. SPSS V21 (IBM, Armonk, NY, USA) was used to conduct the statistical analyses.

## Results

### Intra-group correlations between tests and measuring times

**Fig 3:**
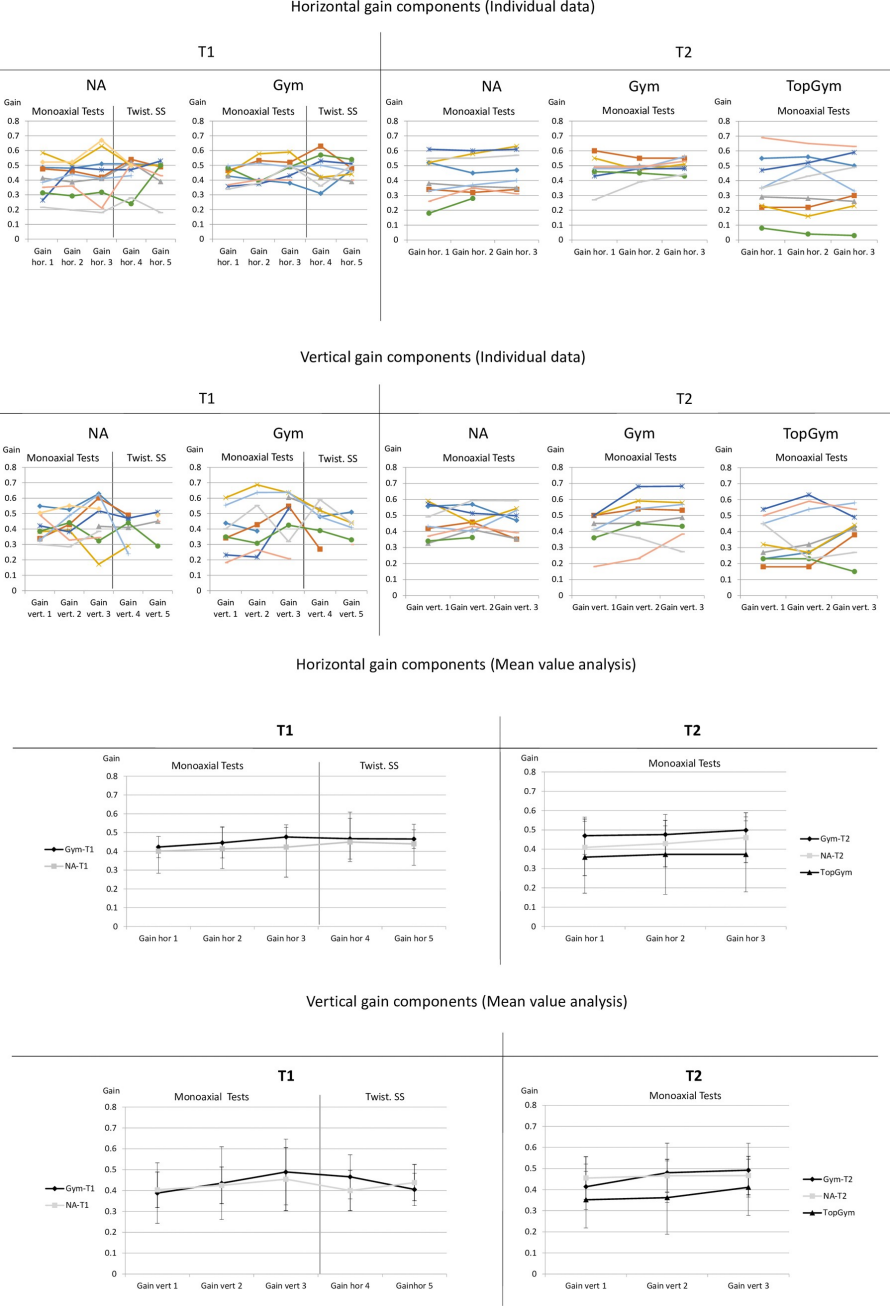
**A–D.** Descriptive data. Horizontal and vertical gains (individual values and mean values +/- 1 SD) over all tests and all groups at T1 and T2. Missing values are mostly caused by eye blink artefacts that did not allow valid data detection. Twist. SS = twisting somersault tests (Tests 4 and 5).

As demonstrated in Fig 3, inter-individual variability of gain values was quite high in all groups. The group of TopGym represented the highest inter-individual range between the subject with lowest values (below 0.1) and the subject with highest values (between 0.6 and 0.7). On the other hand, all groups show a high relative stability of each subjects’ individual gain levels over the different test conditions. This is represented by high correlations between the *monoaxial* test conditions (tests 1–3) in the horizontal and also the vertical plane at both measuring times (horizontal plane: NA at T1: r = 0.81, at T2: r = 0.95; Gym at T1: r = 0.59, at T2: r = 0.78; TopGym (at T2): r = 0.93. vertical plane: NA at T1: r = 0.41, at T2: r = 0.62; Gym at T1: r = 0.75, at T2: r = 0.80; TopGym (at T2): r = 0,70). Even *between* the measurement times (after a period of three years) in the *horizontal plane* valuable intra-individual correlations within the groups were found for gain values (NA T1/T2: r = 0.70, Gym T1/T2: r = 0.41). In the *vertical plane* however correlations between measurement times were lower (NA T1/T2: r = 0.30; Gym T1/T2: r = 0.50).

Concerning the *multiaxial* test conditions, within-group correlations of gain components between the twisting tests (test 4 and 5) were found a bit lower than between *monoaxial* test conditions (tests 1–3) with values in *horizontal* components of NA: r = 0.43; Gym: r = 0.37 and *vertical* components of NA: r = 0.26, Gym: r = 0.57. See also S1 File for more details.

### Differences between tests, groups and measuring times

Within all groups (Gym, NA, TopGym), there was a slight tendency of a horizontal and vertical mean gain increase between tests 1 and 3 (at T1 and T2). However, these differences were not significant. Even though in the Gym group the *original* p-values reached the level of significance in two cases (T1: gain hor 1 vs. gain hor 3 with p = 0.045; T2: gain vert 1 vs. gain vert 2 with p = 0.037), after alpha-correction these differences were not significant. Also concerning the multiaxial tests 4 and 5, no significant differences were found (see also: S1 File).

Concerning *inter-group* differences, the Gym group showed a slight tendency for *higher* gains compared to controls at T1 and T2 (mean difference T1: 0.023 [mean Cohen´s d: 0.279]; mean difference T2: 0.024; [mean Cohen´s d: 0.378]). In contrast, in the group of the TopGym, the *lowest* mean values were found compared to the other groups (mean difference TopGym/Gym at T2: -0.1 [mean Cohen´s d: 0.754]; mean difference TopGym/NA at T2: -0.076 [mean Cohen´s d: 0.564). No inter-group differences were significant.

Concerning differences *between the measuring times* T1 vs. T2, a very slight tendency for an *increase* in both groups was found, but also far below the threshold of significance (mean difference Gym: 0.029 [mean Cohen´s d: 0.332]; mean difference NA: 0.028 [mean Cohen´s d: 0.282]). For more detailed data concerning differences between groups and measuring times see S1 File.

Summarized, there were *no significant differences* of gain values *between test conditions*, *nor between groups*, *nor between measuring times*, neither in horizontal nor vertical monoaxial tests, nor in the multiaxial tests.

## Discussion

### General description and interpretation of the results

The primary aim of the study was to answer whether any differences exist with respect to measures of VOR processing of highly trained gymnasts vs. controls during different types of passively driven rotational stimuli (monoaxial and multi-axial). This question helps provide a better understanding of SCEM (compare: [1]) and how best to functionally interpret it.

#### Monoaxial tests

During monoaxial testing, apart from the well-known, high inter-individual variability in VOR response, a high intra-individual stability could be seen both in non-athletes and in gymnasts, as well. This intra-individual stability was marked by high correlation coefficients between the test conditions and could even be observed between the same tests of T1 compared to T2. This underlines a high, long-term consistency of the individual level of VOR response, even over a time period of three years.

However, the most remarkable result with regard to the primary aim of the study (the question of whether there are differences in the VOR response between gymnasts and non-athletes) was the fact that significant differences between gymnasts and non-athletes were not observed. Even the values in the group of the TopGym did not differ in a consistent way from the Gym and the NA. This might underscore that the VOR gain level is not affected by practicing gymnastics. This result could be consistent with the results of Hartmann et al. [28], who describe no differences in angular motion detection thresholds of (female) gymnasts compared to a non-athlete control group.

An interesting finding in the group of TopGym is a remarkable *separation* of the group into one half representing *high gain values* and four to five athletes with very *low values* (compare Fig 3). This “division” of the sample was found both in the horizontal and vertical plane. Due to the very low VOR gain of four subjects, the group of *TopGym* shows a tendency toward *lower mean values* compared to the other groups, even though the other subjects of the TopGym group show similar and higher values as were found in the groups of Gym and NA. In particular, one athlete in the TopGym group was remarkable in this context, representing *extremely* low gain values (values not observed for any subject of any other group). This might lead to the idea that such an extraordinary low-gain VOR response may either result from practicing gymnastics or that a *naturally* very low (or very high) gain might be a preselection factor associated with sport-specific benefits, such as a higher degree of multiaxial spatial orientation abilities. The coaches who trained the TopGym group were given a questionnaire, asking among other things which gymnast they would estimate as the one with the *highest spatial orientation abilities*. The athlete with the *lowest* VOR-response was chosen as the best one within this group. However, the *second best* gymnast from the questionnaire had the *highest* VOR-response. In summary, the rankings of VOR-levels did not clearly correlate with typical sport-specific abilities as estimated by the coaches—neither with multiaxial spatial orientation aptitudes, nor with other sport-specific abilities. This shows that both very low and also very high gain values are found among the best performers, but no relationship could be found between individual VOR gain levels and context-specific tasks.

Thus, in the context of the other findings of the present study (i.e., no differences between groups), we must interpret the above mentioned finding of such an extraordinarily *low* gain VOR, and also perhaps the large variance of all the TopGym responses, as being due to top-down effects. Differing mindsets of the exact task to be performed due to the novel experience of a unique device may have influenced a motor plan for the response. For such a top-down motor plan (see Interpretations below), differing strategies for performing the task could lead to differing levels of suppression. This may explain the results from the TopGym group, but may not be characteristic for all practicing gymnasts, or associated with the sport-specific level of performance.

Thus, the results do not allow one to safely exclude that individual gain levels might possibly be influenced by practicing gymnastics or may be a factor influencing sport-specific preselection processes. However, it can be summarized that the results give neither evidence nor any indications for the existence of relationships between sport-specific performance and VOR gain levels measured by common standards during passive rotations.

#### Multiaxial tests

The main results of the multiaxial tests did not differ from the monoaxial testing: no significant differences between the tests without prior notice of the type of motion (test 4) and the test after prior notice (test 5), neither in gymnasts nor in non-athletes. Further, there were no significant differences between the groups in both tests. This reinforces the idea that the VOR response does *not* seem to be associated with *more context-specific rotations*, to which gymnasts are intensively accustomed. Even under conditions of mental preparation for that kind of motion, no significant differences compared to non-athletes were found.

**Fig 4:**
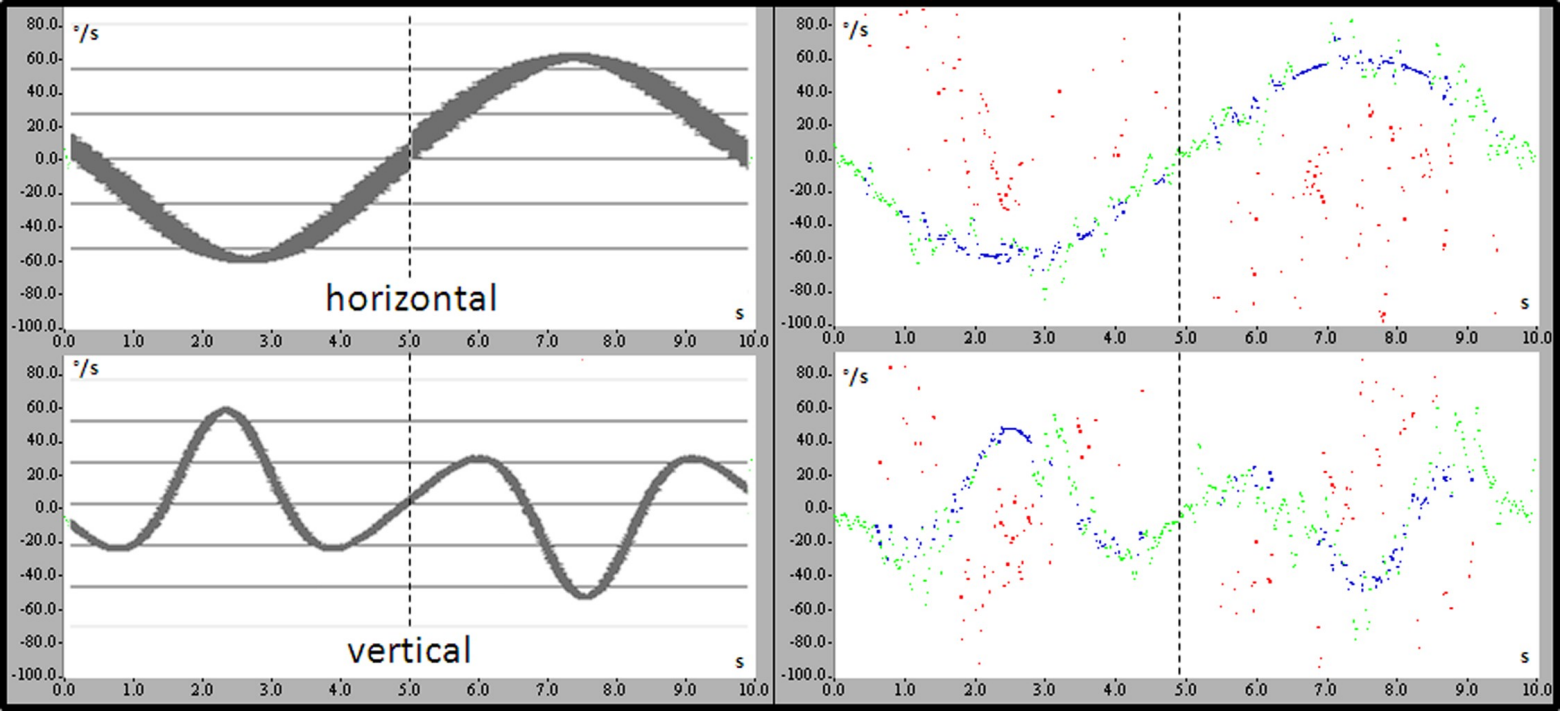
Nystagmus slow phase components during twisting somersaults. Left: Calculated data. Right: Example of original data. The upper graphs each represent the horizontal component (negative values: velocities to the right; positive values: velocities to the left); the lower graphs represent the vertical components (negative values: backward velocities, positive values: forward velocities). The vertical line in the middle of the graphs represents the change of direction of the sinusoidal oscillation. Green colour: original data; blue color: interpolated data; red color: rejected fast components/artifacts.

Viewing the *original data sources* during the multiaxial tests, the slow phase patterns represent the movement of the head exactly in three-dimensional space (Fig 4). These characteristics were likewise represented in *both groups* of subjects (gymnasts and controls) and in *both trials* (with and without prior expectation of the movement).

Putting these observations into the context of the SCEM phenomenon, one can observe that *in contrast* to the “EREM suppression phase”, as observed in real, *active* twisting somersaults (compare Fig 1 in the introduction), during *passive* multiaxial testing the gymnasts (and also the non-athletes) showed *intense nystagmus activity from the very beginning* of the movement. Thus, the sector described as “EREM suppression” (first half of twisting somersaults of gymnasts) was not observed during *passive* twisting movements.

In summary, the nystagmus activity during passive multiaxial testing was strongly associated with the real stimulus components in *both groups* and *no significant differences* in VOR responses between the groups could be detected within *monoaxial* and *multiaxial* tests. The data from the present study do not demonstrate any systematic tendency of adaptative processes for the VOR response in experienced vs. non-experienced subjects during passive rotations.

### Limitations of the present study

We would like to emphasize that our findings only refer to the specific conditions and movement parameters used within the present study (0.1 Hz; with 25, 50 and 100°/s). The tests included well-established “*standard tests*” of monoaxial movments on the one hand (serving as a reference to other publications), and also more “*context specific tests*” of multiaxial movements on the other hand, to specifically evaluate more context-specific adaptations that might not be elucidated by standard tests. In particular, the vertical whole body accelerations and the twisting somersault tests were unique in that manner. Thus, the chosen tests build upon existing protocols to allow for the evaluation of adaptative processes within the expert groups in comparison with the VOR response of the controls, but the reported results only relate to the specific tests used within this study. It cannot be excluded that other tests, in particular tests that approach the goal of context-specificity, might lead to other results. Furthermore, it may be possible that the reported VOR-adaptation of other subject groups (e.g., figure skaters, pilots etc.; compare introduction), may possibly be caused by other *kinds* of stimuli with which these subjects are more familiar (e.g., figure skaters are more accustomed to *long lasting monoaxial rotations* during pirouettes than gymnasts).

Additionally, we emphasize that the results of the comparisons between the TopGym with the other groups need to be interpreted carefully, because this sample was approximately *seven years older* than the samples of Gym and NA. It has been shown that rotational VOR gains in children tend to be higher than in adult [e.g., 29, 30]. Thus, it cannot be excluded with certainty that comparisons between the TopGym with the other groups might be biased by age related factors.

## Conclusions

Based on the well-known ability of the VOR to adapt to external conditions, as has been demonstrated in many prior studies [e.g., 11, 12, 26, 31–35], one might expect changes in VOR parameters caused by adaptative processes in the groups of gymnasts who are highly trained for rotational motions. Additionally, the results of our prior study that demonstrated a complete suppression of nystagmus during specific phases of actively performed twisting somersaults might underscore this expectation (SCEM phenomenon; [1]). However, the present results demonstrate that even though gymnasts show a complete EREM suppression during *real* twisting somersaults (see introduction; [1]), adaptative changes in the VOR *were not* found in high level gymnasts during a passive *simulation* of such movements, even with multiaxial motion and a-prior expectations/preparations.

In our opinion, the present results can be interpreted as generally supporting the conclusion that this complete EREM-suppression phenomenon in gymnasts (SCEM) are likely *not* explained by *adaptative* VOR suppression, rather by a complete *exchange* of eye movements by more *proprioceptively driven functions* [1] or a top-down motor plan which suppresses reflexive responses [36–38] during *real* twisting somersaults. Even though the tests as used in our present study included motions similar to those experienced during gymnastics movements, they were all *passively* driven. So, all intentional and reflexive influences that might be controlling during *actively performed* movements were systematically excluded. Thus, the results of this study seem to support the opinion of Collewijn [39], postulating that VOR-measurements as typically done (passively driven in the dark), may not sufficiently represent the complexity of adaptative eye movement processes during real life conditions. The tight correlation between eye movements and body movements (SCEM), as we have found during *actively* performed twisting somersaults seem to underscore this position.

Thus, the results seem to indicate that the phenomenon of SCEM seems not to be induced by a training-dependent or context-specific *reduction of VOR-functions*, rather that VOR-functions are *replaced* in that phase by situationally dominant, probably *proprioceptively mediated or top-down anti-compensatory* systems. Spatial orientation abilities in gymnasts might therefore be controlled by a context-specific *selection* of the most appropriate or successful systems (instead of a pure *suppression*), to effectively cope with the context specific needs of certain movement phases as we previously postulated [1].

## Supporting information

S1 File Statistical data. (PDF)
