# Supplementary material for: Long term measures of vestibulo-ocular reflex function in high level male gymnasts and its possible role during context specific rotational tasks
Source: PLoS One. 2020 Dec 14;15(12):e0243752. doi: 10.1371/journal.pone.0243752 (PMC7735588; doi:10.1371/journal.pone.0243752)
Supplement: S1 File — (PDF) [file pone.0243752.s001.pdf]

Tab. 1: Descriptive data of all tests and all groups (mono- and multiaxial at T1 and T2)

**VOR-Gain: Mean values ± SD; Coefficient of Variance (CoV)**					
		T1		T2	
	Group	MV ± SD	CoV	MV ± SD	CoV
**Monoaxial**	**Gym**				
Test 1	Gym hor 1	0.423 ± 0.057	0.132	0.470 ± 0.097	0.206
	Gym vert 1	0.388 ± 0.145	0.374	0.414 ± 0.108	0.261
Test 2	Gym hor 2	0.446 ± 0.081	0.182	0.476 ± 0.045	0.095
	Gym vert 2	0.435 ± 0.174	0.400	0.480 ± 0.140	0.292
Test 3	Gym hor 3	0.477 ± 0.064	0.134	0.499 ± 0.048	0.096
	Gym vert 3	0.489 ± 0.158	0.323	0.492 ± 0.128	0.260
**Mean**			**0.258**		**0.202**
**Multiaxial**					
Test 4	Gain hor. 4	0.468 ± 0.109	0.233		
	Gain vert. 4	0.466 ± 0.106	0.227		
Test 5	Gain hor. 5	0.466 ± 0.050	0.107		
	Gain vert. 5	0.405 ± 0.077	0.190		
**Mean**			**0.189**		
**Monoaxial**	**NA**				
Test 1	NA hor 1	0.401 ± 0.118	0.294	0.410 ± 0.146	0.356
	NA vert 1	0.404 ± 0.085	0.144	0.455± 0.101	0.222
Test 2	NA hor 2	0.413 ± 0.104	0.252	0.418 ± 0.135	0.323
	NA vert 2.	0.424 ± 0.083	0.196	0.466 ± 0.078	0.167
Test 3	NA hor 3	0.423 ± 0.160	0.378	0.460 ± 0.132	0.287
	NA vert 3	0.455 ± 0.151	0.332	0.467 ± 0.103	0.221
**Mean**			**0.266**		**0.263**
**Multiaxial**					
Test 4	Gain hor. 4	0.45 ± 0.105	0.233		
	Gain vert. 4	0.4 ± 0.097	0.243		
Test 5	Gain hor. 5	0.44 ± 0.114	0.259		
	Gain vert. 5	0.438 ± 0.087	0.199		
**Mean**			**0.234**		
				(T2)	
**Monoaxial**	**TopGym**				
Test 1	TopGym hor 1	--	--	0.359 ± 0.186	0.518
	TopGym vert 1	--	--	0.352 ± 0.134	0.381
Test 2	TopGym hor 2	--	--	0.373 ± 0.207	0.555
	TopGym vert.2	--	--	0.362 ± 0.174	0.481
Test 3	TopGym hor 3	--	--	0.373 ± 0.194	0.520
	TopGym vert 3	--	--	0.411 ± 0.133	0.324
**Mean**					**0.410**

Tab. 2: Correlations between test conditions within the groups (NA, Gym at T1 and T2; TopGym)

	**Correlations between test conditions within the groups at T1 and T2**<br>VOR-Gain: Pearson correlation coefficients, Fisher´s Z, Inverted Fisher´s Z									
	**T1**				**T2**				**(T2)**	
**Monoaxial**	**NA T1**		**Gym T1**		**NA T2**		**Gym T2**		**TopGym**	
	Pearson	Z	Pearson	Z	Pearson	Z	Pearson	Z	Pearson	Z
Gain hor. 1/2	0.740	0.949	0.598	0.689	0.927	1.640	0.879	1.371	0.944	1.772
Gain vert. 1/2	0.457	0.494	0.911	1.533	0.676	0.821	0.861	1.296	0.824	1.170
Gain hor. 2/3	0.878	1.368	0.728	0.924	0.983	2.367	0.721	0.909	0.919	1.582
Gain vert. 2/3	0.557	0.629	0.514	0.567	0.482	0.525	0.874	1.350	0.727	0.921
Gain hor. 1/3	0.789	1.069	0.378	0.397	0.914	1.550	0.674	0.818	0.917	1.570
Gain vert. 1/3	0.171	0.173	0.687	0.843	0.673	0.816	0.583	0.667	0.490	0.536
Mean Z hor		1.129		0.67		1.852		1.032		1.641
**Inv Fish. hor**	0.811		0.585		0.952		0.775		0.928	
Mean Z vert		0.432		0.981		0.721		1.104		0.876
**Inv Fish. vert**	0.407		0.753		0.617		0.802		0.704	
	**T1 / T2**									
	**NA T1/T2**		**Gym T1/T2**							
	Pearson	Z	Pearson	Z						
Gain hor. 1	0.346	0.361	0.631	0.743						
Gain vert. 1	0.382	0.402	0.470	0.510						
Gain hor. 2	0.800	1.097	0.502	0.552						
Gain vert. 2	0.507	0.559	0.165	0.166						
Gain hor. 3	0.809	1.124	0.020	0.020						
Gain vert. 3	-0.031	-0.031	0.747	0.966						
Mean Z hor		0.861		0.438						
**Inv Fish. hor**	0.697		0.412							
Mean Z vert		0.310		0.547						
**Inv Fish. vert**	0.300		0.499							
	**T1**									
**Multiaxial**	**NA T1**		**Gym T1**							
	Pearson		Pearson							
Gain hor. 4/5	0.431		0.371							
Gain vert. 4/5	0.264		0.569							

Tab. 3: Differences between test conditions within the groups (Gym and NA at T1 and T2; TopGym)

**Differences between test conditions within the groups at T1 and T2**<br>VOR-Gain: Mean values (MV) ± Standard deviation (SD); Cohen´s d; Original p-values								
	**Gym**							
	**T1**							
**Monoaxial**	MV	SD	MV	SD	Diff. MV	Mean SD	Cohen´s d	p- value
Gain hor. 1/2	0.423	0.057	0.446	0.081	-0.023	0.069	0.333	0.352
Gain vert. 1/2	0.388	0.145	0.435	0.174	-0.047	0.160	0.294	0.108
Gain hor. 2/3	0.446	0.081	0.477	0.064	-0.031	0.073	0.425	0.189
Gain vert. 2/3	0.435	0.174	0.489	0.158	-0.054	0.166	0.325	0.664
Gain hor. 1/3	0.423	0.057	0.477	0.064	-0.054	0.061	0.885	0.045
Gain vert. 1/3	0.388	0.145	0.489	0.158	-0.101	0.152	0.134	0.102
**Monoaxial vs. Multiaxial**								
Gain hor. 3/4	0.477	0.064	0.468	0.109	0.009	0.087	0.103	0.643
Gain vert. 3/4	0.489	0.158	0.466	0.106	0.023	0.132	0.174	0.427
Gain hor. 3/5	0.477	0.064	0.466	0.050	0.011	0.057	0.193	0.687
Gain vert. 3/5	0.489	0.158	0.405	0.077	0.084	0.118	0.712	0.444
**Multiaxial**								
Gain hor. 4/5	0.468	0.109	0.466	0.050	0.002	0.080	0.025	0.866
Gain vert. 4/5	0.466	0.106	0.405	0.077	0.061	0.092	0.663	0.083
	**T2**							
**Monoaxial**	MV	SD	MV	SD	Diff. MV	Mean SD	Cohen´s d	p- value
Gain hor. 1/2	0.470	0.097	0.476	0.045	-0.006	0.071	0.085	0.780
Gain vert. 1/2	0.414	0.108	0.480	0.140	-0.066	0.124	0.532	0.037
Gain hor. 2/3	0.476	0.045	0.499	0.048	-0.023	0.047	0.489	0.111
Gain vert. 2/3	0.480	0.140	0.492	0.128	-0.012	0.134	0.090	0.632
Gain hor. 1/3	0.470	0.097	0.499	0.048	-0.029	0.073	0.392	0.306
Gain vert. 1/3	0.414	0.108	0.492	0.128	-0.078	0.118	0.661	0.081
	**NA**							
	**T1**							
**Monoaxial**	MV	SD	MV	SD	Diff. MV	Mean SD	Cohen´s d	p- value
Gain hor. 1/2	0.401	0.118	0.413	0.104	-0.012	0.111	0.108	0.646
Gain vert. 1/2	0.404	0.085	0.424	0.083	-0.02	0.084	0.238	0.670
Gain hor. 2/3	0.413	0.104	0.423	0.160	-0.01	0.132	0.076	0.730
Gain vert. 2/3	0.424	0.083	0.455	0.151	-0.031	0.117	0.265	0.464
Gain hor. 1/3	0.401	0.118	0.423	0.160	-0.022	0.139	0.158	0.503
Gain vert. 1/3	0.404	0.085	0.455	0.151	-0.051	0.118	0.432	0.341
**Monoaxial vs. Multiaxial**								
Gain hor. 3/4	0.423	0.160	0.45	0.105	-0.027	0.133	0.203	0.549
Gain vert. 3/4	0.455	0.151	0.4	0.097	0.055	0.124	0.444	0.337
Gain hor. 3/5	0.423	0.160	0.44	0.114	-0.017	0.137	0.124	0.284
Gain vert. 3/5	0.455	0.151	0.438	0.087	0.017	0.119	0.143	0.702
**Multiaxial**								
Gain hor. 4/5	0.45	0.105	0.44	0.114	0.01	0.110	0.091	0.890
Gain vert. 4/5	0.4	0.097	0.438	0.087	-0.038	0.092	0.413	0.748
	**T2**							
**Monoaxial**	MV	SD	MV	SD	Diff. MV	Mean SD	Cohen´s d	p- value
Gain hor. 1/2	0.41	0.146	0.429	0.120	-0.019	0.133	0.143	0.348
Gain vert. 1/2	0.455	0.101	0.466	0.078	-0.011	0.090	0.122	0.676
Gain hor. 2/3	0.429	0.120	0.46	0.129	-0.031	0.125	0.248	0.234
Gain vert. 2/3	0.466	0.078	0.467	0.091	-0.001	0.085	0.012	0.694

Gain hor. 1/3	0.41	0.146	0.46	0.129	-0.05	0.138	0.362	0.294
Gain vert. 1/3	0.455	0.101	0.467	0.091	-0.012	0.096	0.125	0.922
	**TopGym (T2)**							
**Monoaxial**	MV	SD	MV	SD	Diff. MV	Mean SD	Cohen´s d	p- value
Gain hor. 1/2	0.359	0.186	0.373	0.207	-0.014	0.197	0.071	0.546
Gain vert. 1/2	0.352	0.134	0.362	0.174	-0.01	0.154	0.065	0.770
Gain hor. 2/3	0.373	0.207	0.373	0.194	0	0.201	0	1
Gain vert. 2/3	0.362	0.174	0.411	0.133	-0.049	0.154	0.318	0.255
Gain hor. 1/3	0.359	0.186	0.373	0.194	0.014	0.190	0.074	0.593
Gain vert. 1/3	0.352	0.134	0.411	0.133	-0.059	0.134	0.440	0.227

Tab. 4: Longitudinal analysis T1 vs. T2 (Gym and NA)

**Longitudinal analysis: T1 vs. T2 (Gym and NA)**<br>VOR-Gain: Mean values (MV) ± Standard deviation (SD); Cohen´s d; Original p-values									
		**Gym**							
		**T1**		**T2**					
		MV	SD	MV	SD	Diff. MV	Mean SD	Cohen´s d	p- value
**Monoaxial**									
Test 1	Gain hor. 1	0.423	0.057	0.470	0.097	-0.047	0.077	0.610	0.118
	Gain vert. 1	0.388	0.145	0.414	0.108	-0.026	0.127	0.205	0.624
Test 2	Gain hor. 2	0.446	0.081	0.476	0.045	-0.03	0.063	0.476	0.493
	Gain vert. 2	0.435	0.174	0.480	0.140	-0.045	0.157	0.287	0.632
Test 3	Gain hor. 3	0.477	0.064	0.499	0.048	-0.022	0.056	0.393	0.712
	Gain vert. 3	0.489	0.158	0.492	0.128	-0.003	0.143	0.021	0.933
	Mean hor	0.449	0.067	0.482	0.063			0.493	
	Mean vert	0.437	0.159	0.462	0.125			0.171	
	**Mean**	**0.443**	**0.113**	**0.472**	**0.097**	**-0.029**	**0.105**	**0.332**	
		**NA**							
		**T1**		**T2**					
		MV	SD	MV	SD	Diff. MV	Mean SD	Cohen´s d	p- value
**Monoaxial**									
Test 1	Gain hor. 1	0.401	0.118	0.41	0.146	-0.009	0.132	0.068	0.818
	Gain vert. 1	0.404	0.085	0.455	0.101	-0.051	0.093	0.548	0.279
Test 2	Gain hor. 2	0.413	0.104	0.429	0.120	-0.016	0.112	0.143	0.744
	Gain vert. 2	0.424	0.083	0.466	0.078	-0.042	0.081	0.519	0.300
Test 3	Gain hor. 3	0.423	0.160	0.46	0.129	-0.037	0.118	0.314	0.842
	Gain vert. 3	0.455	0.151	0.467	0.091	-0.012	0.121	0.099	0.848
	Mean hor	0.412	0.127	0.433	0.132			0.175	
	Mean vert	0.428	0.106	0.463	0.09			0.389	
	**Mean**	**0.42**	**0.117**	**0.448**	**0.111**	**-0.028**	**0.114**	**0.282**	

Tab. 5: Cross-sectional analysis Gym vs. NA (at T1 and T2)

**Cross-sectional analysis NA vs. Gym (at T1 and T2)**<br>VOR-Gain: Mean values (MV) ± Standard deviation (SD); Cohen´s d; Original p-values									
		**T1**							
		**NA T1**		**Gym T1**					
		MV	SD	MV	SD	Diff. MV	Mean SD	Cohen´s d	p-value
**Monoaxial**									
Test 1	Gain hor. 1	0.401	0.118	0.423	0.057	-0.022	0.087	0.253	0.617
	Gain vert. 1	0.404	0.085	0.388	0.145	0.016	0.118	0.136	0.777
Test 2	Gain hor. 2	0.413	0.104	0.446	0.081	-0.033	0.093	0.355	0.480
	Gain vert. 2	0.424	0.083	0.435	0.174	-0.011	0.129	0.085	0.871
Test 3	Gain hor. 3	0.423	0.160	0.477	0.064	-0.054	0.112	0.482	0.359
	Gain vert. 3	0.455	0.151	0.489	0.158	-0.056	0.155	0.361	0.644
	Mean hor	0.412	0.127	0.449	0.067			0.363	
	Mean vert	0.428	0.106	0.437	0.159			0.194	
	**Mean**	**0.42**	**0.117**	**0.443**	**0.113**	**-0.023**	**0.115**	**0.279**	
**Multiaxial**									
Test 4	Gain hor. 4	0.45	0.105	0.468	0.109	-0.018	0.107	0.168	0.734
	Gain vert. 4	0.4	0.097	0.466	0.106	-0.066	0.102	0.647	0.248
Test 5	Gain hor. 5	0.44	0.114	0.466	0.050	-0.026	0.082	0.317	0.550
	Gain vert. 5	0.438	0.087	0.405	0.077	0.033	0.082	0.402	0.522
	Mean hor	0.445	0.110	0.467	0.080			0.243	
	Mean vert	0.419	0.092	0.436	0.092			0.525	
	**Mean**	**0.432**	**0.101**	**0.452**	**0.086**	**-0.02**	**0.094**	**0.384**	
		**T2**							
		**NA T2**		**Gym T2**					
		MV	SD	MV	SD	Diff. MV	Mean SD	Cohen´s d	p-value
**Monoaxial**									
Test 1	Gain hor. 1	0.41	0.146	0.470	0.097	-0.06	0.121	0.496	0.342
	Gain vert. 1	0.455	0.101	0.414	0.108	0.041	0.100	0.41	0.430
Test 2	Gain hor. 2	0.429	0.120	0.476	0.045	-0.047	0.083	0.566	0.312
	Gain vert. 2	0.466	0.078	0.480	0.140	-0.014	0.109	0.128	0.796
Test 3	Gain hor. 3	0.46	0.129	0.499	0.048	-0.039	0.089	0.438	0.439
	Gain vert. 3	0.467	0.091	0.492	0.128	-0.025	0.110	0.227	0.651
	Mean hor	0.433	0.132	0.482	0.063			0.5	
	Mean vert	0.463	0.09	0.462	0.125			0.255	
	Mean	**0.448**	**0.111**	**0.472**	**0.097**	**-0.024**	**0.104**	**0.378**	

Tab. 6: Comparisons TopGym vs. Gym and NA (at T1 and T2)

**Comparisons TopGym vs. Gym and NA (at T1 and T2)**<br>VOR-Gain: Mean values (MV) ± Standard deviation (SD); CoV, Cohen´s d; Original p-values									
		MV	SD	MV	SD	Diff. MV	Mean SD	Cohen´s d	p-value
		**TopGym**		**Gym at T1**					
**Monoaxial**									
Test 1	Gain hor. 1	0.359	0.186	0.423	0.057	-0.064	0.122	0.525	0.337
	Gain vert. 1	0.352	0.134	0.388	0.145	-0.036	0.140	0.257	0.607
Test 2	Gain hor. 2	0.373	0.207	0.446	0.081	-0.073	0.144	0.507	0.368
	Gain vert. 2	0.362	0.174	0.435	0.174	-0.073	0.174	0.420	0.401
Test 3	Gain hor. 3	0.373	0.194	0.477	0.064	-0.104	0.129	0.806	0.149
	Gain vert. 3	0.411	0.133	0.489	0.158	-0.078	0.146	0.534	0.288
	Mean hor	0.368	0.195	0.449	0.067			0.613	
	Mean vert	0.375	0.147	0.437	0.159			0.405	
	**Mean**	**0.372**	**0.171**	**0.443**	**0.113**			**0.508**	
		**TopGym**		**NA at T1**					
**Monoaxial**									
Test 1	Gain hor. 1	0.359	0.186	0.401	0.118	-0.042	0.143	0.294	0.558
	Gain vert. 1	0.352	0.134	0.404	0.085	-0.052	0.110	0.473	0.328
Test 2	Gain hor. 2	0.373	0.207	0.413	0.104	-0.04	0.156	0.256	0.595
	Gain vert. 2	0.362	0.174	0.424	0.083	- 0.062	0.102	0.608	0.352
Test 3	Gain hor. 3	0.373	0.194	0.423	0.160	-0.05	0.177	0.282	0.550
	Gain vert. 3	0.411	0.133	0.455	0.151	-0.044	0.142	0.310	0.518
	Mean hor	0.368	0.195	0.412	0.127			0.277	
	Mean vert	0.375	0.147	0.428	0.106			0.464	
	**Mean**	**0.372**	**0.171**	**0.42**	**0.117**			**0.371**	
		**TopGym**		**Gym at T2**					
**Monoaxial**									
Test 1	Gain hor. 1	0.359	0.186	0.470	0.097	-0.111	0.142	0.782	0.151
	Gain vert. 1	0.352	0.134	0.414	0.108	-0.062	0.121	0.512	0.317
Test 2	Gain hor. 2	0.373	0.207	0.476	0.045	-0.103	0.126	0.817	0.189
	Gain vert. 2	0.362	0.174	0.480	0.140	-0.118	0.157	0.752	0.149
Test 3	Gain hor. 3	0.373	0.194	0.499	0.048	-0.126	0.121	1.041	0.096
	Gain vert. 3	0.411	0.133	0.492	0.128	-0.081	0.131	0.618	0.221
	Mean hor	0.368	0.195	0.482	0.063			0.88	
	Mean vert	0.375	0.147	0.462	0.125			0.627	
	**Mean**	**0.372**	**0.171**	**0.472**	**0.097**			**0.754**	
		**TopGym**		**NA at T2**					
**Monoaxial**									
Test 1	Gain hor. 1	0.359	0.186	0.41	0.146	-0.051	0.166	0.307	0.526
	Gain vert. 1	0.352	0.134	0.455	0.101	-0.103	0.118	0.873	0.085
Test 2	Gain hor. 2	0.373	0.207	0.429	0.120	-0.056	0.164	0.341	0.495
	Gain vert. 2	0.362	0.174	0.466	0.078	-0.104	0.126	0.825	0.123
Test 3	Gain hor. 3	0.373	0.194	0.46	0.129	-0.087	0.162	0.537	0.302
	Gain vert. 3	0.411	0.133	0.467	0.091	-0.056	0.112	0.500	0.339
	Mean hor	0.368	0.195	0.433	0.132			0.395	
	Mean vert	0.375	0.147	0.463	0.09			0.733	
	**Mean**	**0.372**	**0.171**	**0.448**	**0.111**			**0.564**	
